# The Importance of Cancer Stem Cells and Their Pathways in Endometrial Cancer: A Narrative Review

**DOI:** 10.3390/cells14080594

**Published:** 2025-04-14

**Authors:** Laura Georgiana Caravia, Melinda Ildiko Mitranovici, Ioan Emilian Oala, Andreea Taisia Tiron, Anca Angela Simionescu, Alina Maria Borcan, Marius Craina

**Affiliations:** 1 Division of Cellular and Molecular Biology and Histology, Department of Morphological Sciences, “Carol Davila” University of Medicine and Pharmacy, 050474 Bucharest, Romania; laura.caravia@umfcd.ro; 2 Public Health Department, “George Emil Palade” University of Medicine, Pharmacy, Sciences and Technology, 540142 Targu Mures, Romania; 3 Department of Obstetrics and Gynecology, Emergency County Hospital Hunedoara, 14 Victoriei Street, 331057 Hunedoara, Romania; oalaioanemilian@gmail.com; 4 Faculty of Medicine, “Carol Davila” University of Medicine and Pharmacy, 050474 Bucharest, Romania; andreea.tiron@umfcd.ro; 5 Department of Obstretics and Gynecology, Filantropia, Faculty of Medicine Carol Davila, 011171 Bucharest, Romania; anca.simionescu@umfcd.ro; 6 Department of Microbiology, National Institute for Infectious Diseases “Prof. Dr. Matei Balș”, Faculty of Medicine Carol Davila, 021105 Bucharest, Romania; alina.borcan@umfcd.ro; 7 Department of Obstetrics and Gynecology, “Victor Babes” University of Medicine and Pharmacy Timisoara, 300041 Timisoara, Romania; craina.marius@umft.ro

**Keywords:** endometrial cancer, cancer stem cells, signaling stemness pathways, stemness therapy

## Abstract

Endometrial cancer is one of the most common malignancies seen in women in developed countries. While patients in the early stages of this cancer show better responses to surgery, adjuvant hormonal therapy, and chemotherapy, patients with recurrence show treatment resistance. Researchers have recently focused on cancer stem cells (CSCs) in the treatment of gynecologic cancer in general but also specifically in endometrial cancer. CSCs have been investigated because of their resistance to conventional therapies, such as chemo- and radiotherapy, and their ability to induce the progression and recurrence of malignancy. The activation of alternative pathways, such as WNT, PI3K, NF-kB, or NOTCH, could be the basis of the acquisition of these abilities of CSCs. Their specific markers and signaling pathways could be treatment targets for CSCs. In this article, we discuss the importance of obtaining a better understanding of the molecular basis and pathways of CSCs in endometrial cancer and the role of CSCs, aiming to discover more specific therapeutic approaches.

## 1. Introduction

The most common gynecological cancer is endometrial cancer, which is cancer stem cell (CSC)-driven [1]. In patients with advanced disease, the development of resistance is typical. In this context, as endometrial CSCs are involved in invasiveness and metastasis, understanding their role is of great importance in developing a management strategy based on how they could be targeted [1]. Recurrence is relatively frequent, with a prevalence of 13% in high-risk patients [2]. The five-year overall survival rate drops from 95% in stage I to 15–17% in stage III-IV [3] due to poor responses to standard endometrial cancer therapy [4,5].

The CSC theory was developed in studies by Virchow and Cohnheim [2,6,7]. CSCs play a major role in initiation, cancer growth, and progression, and they can self-renew since they are highly tumorigenic cells [2,7]. The interactions of CSCs with the tumor microenvironment promote mutations in which inflammation and oxidative stress are involved. This complex process allows for their own survival, resistance to therapy, and metastatic capability, and they can also migrate freely throughout different tissues [1,2]. Therefore, CSCs are potential therapeutic targets [7]. Plasticity, which is the capability of an organism to undergo a phenotypic modification in the case of stimulation, allows CSCs to be connected to the microenvironment along with differentiated cells due to genomic instability [1].

To develop targeted treatment and molecular diagnosis strategies, biomarkers have been investigated by researchers. Several markers have been used as surface markers for the isolation of CSCs, such as CD133, aldehyde dehydrogenase (ALDH), CD44, and LY6A, but their use is still controversial [1,2,7,8]. There is also no strong evidence that any of these markers are universally specific for endometrial cancer stem cells [2]. Genomic and proteomic profiling can provide more data to enlighten us about the molecular biology of EC and the role of CSCs [2].

Stemness pathways have also been studied, with the most common activated pathways in endometrial CSCs found being Wingless-INT (Wnt)/β-catenin, Hedgehog, and Notch1. These pathways are linked to the proteins involved in self-renewal, such as octamer-binding transcription factor 4 (OCT), North American Network Operations Group Homebox protein (NANOG), and SRY-Box 2 (SOX2). The goals in detecting and isolating these markers of CSCs are to develop a personalized management strategy based on targeting these markers or pathways and to eradicate endometrial cancer [1,2,7].

The aim of our review is to identify some stemness pathways and their clinical significance for the development of personalized treatment for endometrial cancer.

## 2. Materials and Methods

We used the PubMed, Google Scholar, Web of Science, and cBioPortal databases to select relevant studies for our narrative review, using the specific keywords “endometrial cancer”,” cancer stem cells”, “signaling stemness pathways”, and “stemness therapy”. An extensive literature review was conducted, including articles published between 2005 and 2025. PubMed Advanced Search Builder was used to examine titles and abstracts for an adequate selection of references. The inclusion criteria were full-text articles written in English, as well as the clarity and informativeness of the information presented. The exclusion criteria were studies written in languages other than English, books, editorials, and studies with an inappropriate design or those not aligned with the aim of our review. We decided, based on the heterogeneity of the data, that the most appropriate type of review for our study starting with the main question and the aim of our research, is a narrative review.

We explored the idea of a scoping review, which generates hypotheses, and then excluded a systematic review, which focuses on a narrower niche, analyzing, for example, a single important pathway and its relevance for endometrial cancer, and determined that a rigorous search of data from the literature is required. However, we also turned to a narrative review based on the descriptive elements obtained from the keywords; in a systematic review, we would need to answer more analytical questions.

From 2546 titles that were identified through our search, and after duplicates were removed, we had a total of 128 relevant articles. This is due to a manual selection made by two independent authors, starting mainly from the articles containing the most common pathways involved in endometrial cancer which were considered suitable, and a rigorous evaluation.

**Figure 1:**
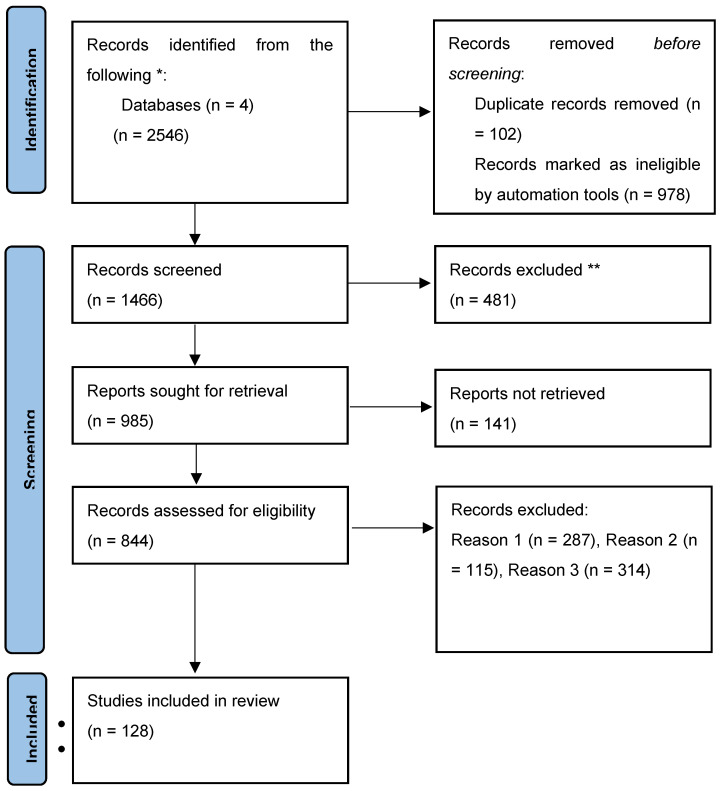
Flow diagram with selection mode.* Number of records identified from Google Scholar, Web of Science, cBio-Portal, and PubMed databases. ** Records were manually excluded. Reason 1: Records were excluded since they were published in languages other than English. Reason 2: Records were excluded by reviewer due to inaccurate or inappropriate titles. Reason 3: Records were excluded based on this study’s research design.

A flow diagram [9] is presented in Figure 1 to show the article selection process.

## 3. Histology Classification of Endometrial Cancer and Stemness

Historically, EC has been divided into two histo-pathogenetic types—endometrioid (Type 1) and non-endometrioid carcinomas (Type 2)—according to Bokhman’s model, which is based on clinical and hormonal features [10]. Type 1 has low potential for lympho-vascular invasion, is characterized by high estrogen dependence with the expression of estrogen and progesterone receptors, and has a favorable prognosis [10]. Type 2 has high potential for lympho-vascular invasion, low progesterone receptor expression, and an unfavorable prognosis [1,2,10].

Molecular categorization changed the staging system for endometrial cancer, which was published in 2023, including tumor patterns and diverse histological classifications [11].

A molecular classification was proposed by The Cancer Genome Atlas (TCGA), defining four EC types based on the mutations encountered, including polymerase epsilon (POLE), p53, and phosphatase and TENsin homolog (PTEN) mutations, as well as histology and microsatellite instability (MSI) [3,12], each with different management and different outcomes [1,13].

### Stem Cells and Endometrium

In a normal human endometrium, populations of epithelial and stromal stem colony-forming cells were found with exceptional regenerative capacity [14,15]. Endometrium suffers monthly regeneration under the influence of ovarian steroid hormones. This process is represented by the cycle of proliferation and differentiation, followed by shedding and regeneration. Endometrium regeneration also allows the uterus to be adjusted for fetus development during pregnancy [2,16,17].

There are four types of stem cells. Totipotent stem cells have the highest differentiation potential; they can generate cells for an entire organism. Pluripotent stem cells are typically embryonic stem cells (ESCs), and they can form germ layer cells. Extraembryonic tissues are not formed via pluripotent stem cells. Multipotent stem cells, such as hematopoietic stem cells, can differentiate into different cell types from a specific cell lineage. Unipotent stem cells possess restricted differentiation capabilities, and they have the unique ability to repeatedly divide, which is why they can be used in regenerative medicine development [2,18].

Cancer stem cells have features similar to those of normal stem cells but with highly tumorigenic activity characterized by aggressiveness; invasion; unlimited proliferation; recurrence; metastasis; self-renewal; chemo-, radio-, and endocrine resistance; and multipotential differentiation, which are seen in 0.02–0.08% of cells in endometrial cancer cell lines [2,19].

## 4. Pathways in Endometrial Cancer Stemness

Different pathways are involved in stemness in EC, among which the Wnt, Notch, and Hedgehog pathways play the most important roles [1].

The theory of cancer stem cells (CSCs) proposes that there is a single subpopulation of cells, namely, CSCs, that have self-regeneration and high differentiation capacities; these cells are the origin for different cells within a tumor and in metastasis. Alternative pathways play a role in the acquisition of these abilities. The WNT, NOTCH, PI3K, TGF-β, NF-κB, and Hippo pathways are the most important pathways identified as being a part of the process.

### 4.1. Hippo 

Hippo is responsible for apoptotic resistance and regulating the autophagy pathway and detoxification mechanisms. It also activates specific stem cell transcription factors and releases drug transporter proteins [20,21,22].

### 4.2. Nanog

Nanog is a key transcription factor that is situated on chromosome 12. It contributes to the preservation of the dormant state of pluripotent stem cells. Cell differentiation can be induced by Nanog downregulation as it is involved in EC [23]. Transcription factors octamer-binding transcription factor 4 (Oct4), transcription factor 3 (Tcf3), SRY-Box 2 (SOX2), and Forkhead Box D3 (FoxD3) are present in Nanog’s expression. OCT-4 and SOX-2 were found to be linked to endometrial cancer stem cells’ (CSCs) self-renewal capacity [1,23].

### 4.3. Wingless Int-1 Wnt Signaling 

Wnt signaling includes three different pathways: the canonical, non-canonical beta-independent, and non-canonical Wnt/calcium pathways [24]. The Wnt/β-catenin canonical pathway, which disrupts β-catenin [8], is involved in embryonic development and adult tissue homeostasis, along with the Hedgehog (Hh) pathway [1,25]. Wnt ligands initiate intracellular signaling via β-catenin nuclear translocation after binding to Frizzled receptors as they are one of the more than nineteen ligands involved in the Wnt signaling cascade [1,20,26]. Kusanoki et al. showed that the downregulation of Wnt signals in endometrial cancer can limit the proliferation, invasion, and metastasis of endometrial cancer stem cells CSCs [27] as they are critical for maintaining stemness [1,17]. β-catenin mutations lead to the downregulation of Wnt antagonists via epigenetic silencing, which is linked to estrogen and progesterone [28]. Estrogen and progesterone receptors are also influenced by mTOR and Hedgehog signaling, which is a challenge in discovering new efficient therapy strategies in EC [28]. Wnt has been demonstrated to participate in cross-talk with the Hedgehog and Notch pathways, which comes with clinical implications in terms of finding new therapeutic options in several cancers without damaging normal somatic cells [25]. Beta-catenin-mediated Wnt signaling regulates the Notch target gene Hes1 [25]. In Wnt’s initiation, Axin plays an important role, which also leads to the membrane destabilization of the beta–catenin complex [25].

### 4.4. NF-κB Pathway

The NF-κB pathway, which has an important role in cellular proliferation and differentiation, has been studied for its roles in inflammation and immune responses. The modulation of cancer is associated with NF-κB’s inflammatory effects [29,30], which lead to genetic alterations in different cancer cells. NF-κB causes the secretion of cytokines and chemokines, such as IL-6, IL8B, and TNF [20,30,31,32].

### 4.5. Notch Pathway 

The Notch pathway is a signaling route involved in many homeostatic processes, such as stem cell maintenance, proliferation, differentiation, and angiogenesis [33,34,35]. Notch functions as an oncogene, and its deregulation contributes to different diseases, such as cancers, viral infections, and congenital malformations [20,34]. This pathway is also implicated in many mechanisms as it regulates cell differentiation in embryos, and in adulthood, it can preserve the dormant state of undifferentiated cells [1,36].

### 4.6. Hedgehog (Hh)

The Hedgehog pathway is normally responsible for embryonic development and organ and tissue homeostasis [20,37], but in the case of abnormal activation, it can be involved in the evolutions of several cancers [20,38,39] through its effects on cell fate determination, the epithelial-to-mesenchymal transition, cell proliferation, and adhesion [1,20,40]. However, there are currently no Sonic Hedgehog (SHH) pathway elements included in the panels of prognostic molecular patterns in EC or drugs targeting this pathway that have been approved for therapy [41].

### 4.7. The TME, Oxidative Stress

The oxidative stress in the tumor microenvironment (TME), hypoxia, and oxidative stress have important, but not completely clear, roles in the dedifferentiation of cells [42]. This process could represent a further event in cancer initiation. Factors promoting Notch activation or the hypoxic microenvironment are linked to the self-renewal ability of stem and non-stem cells. CSCs can suffer from undergoing an abnormal differentiation process, and they can also acquire stem-like properties through stemness signaling pathways, such as Nanog, Oct4, and Sox2, with miRNA also being involved [42,43].

A high concentration of reactive oxygen species (ROS) is detrimental to cells, as ROS promote DNA and RNA modifications and protein and lipid alterations [20,44,45]. Oxidative stress occurs due to cancer cells’ active metabolism, which is induced by altered oncogenes and tumor suppressor signaling pathways. However, cancer cells become resistant to oxidative stress [20,46,47,48]. ROS have been associated with cell proliferation, invasion, metastasis, apoptosis evasion, and angiogenesis. ROS are associated with different signaling pathways, such as the NF-kB signaling pathway and Wnt signaling cascade stimulation [20,45]. Aerobic glycolysis is responsible for the generation of a lot of ROS, and it is energetically more efficient than the anaerobic route, but cancer cells are adapting to oxidative stress by switching from aerobic to anaerobic glycolysis. This is referred to as the Warburg effect and is independent of the oxygen level available in the microenvironment. This phenomenon leads to a low ROS formation level through NADPH production [20,46,47]. Furthermore, CSCs have higher antioxidant efficiency compared with normal cells [1,20,48].

### 4.8. The Epithelial–Mesenchymal Transition (EMT) 

EMT is a process related to stemness in ECs but is under-investigated [1]. It is a molecular process of reprogramming immobile and polarized epithelial cells into mobile mesenchymal cells, and it is also involved in invasion and metastases [49,50]. During this process, a decrease is observed in the expression levels of adhesion molecules, such as γ-catenin and E-cadherin, and an increase is observed in the levels of mesenchymal markers (vimentin, fibronectin, and N-cadherin) and extracellular matrix metalloproteinases [51,52,53]. The EMT is an important process in the tumor microenvironment (TME). An extremely hypoxic environment can lead to the selection of more aggressive CSC tumor cells, which are more likely to survive and proliferate [54]. Hypoxia is mediated by HIFs, which are important sensors of intracellular oxygen alterations [55] and are activated via Notch signaling, which is the key regulating pathway for the hypoxia response [54,56].

### 4.9. The PTEN/Phosphoinositide 3 Kinase (PI3K)/Protein Kinase B (AKT)/Mammalian Target of Rapamycin (mTOR) Pathway 

PI3K/mTOR pathway plays a key role in maintaining the stemness associated with the upregulation of EMT inducers. Some of these inducers include B Lymphoma Mo-MLV Insertion Region 1 Homolog (BMI-1), which is involved in PTEN downregulation, and enhancer of zeste homolog 2 (EZH2), which is involved in E-cadherin downregulation through histone methylation [1,57]. The interplay between CSCs and the epithelial–mesenchymal transition (EMT) drives cancer progression, metastasis, and therapy failure. The involvement of epigenetic deregulation in these processes has been investigated. The methylation of histone and non-histone proteins is recognized as deregulated in many cancers and is associated with the EMT and cellular plasticity, leading to therapy resistance [58].

### 4.10. Associated Factors Linked to Stemness Pathways

A myriad of compounds targeting CSCs have been developed based on signaling pathways [1]. The subset of cells with stem cell-like properties is essential in tumorigenesis because of their self-renewal ability [1,8,59] as they have a critical role in the EMT process and drug resistance [8,57,60,61]. There is an important relationship between EC and metabolic disorders, and the overexpression of lizophosphatidylcholine acyltransferase 1 (LPCAT1) is linked to stemness enhancement and metastasis in endometrial cancer. This is also observed in younger women. The contents of various phospholipids, such as phosphatidylcholine (PC) and triglyceride (TG), are changed via the overexpression of LPCAT1. LPCAT1 promotes the overexpression of EMT-related proteins through the TGF-β/Smad2/3 signaling pathway [62].

Researchers have focused on the correlation between gene expression and stemness pathways when searching for new molecular markers for targeted treatments and molecular diagnostic tools. MiRNA, an epigenetic modulator, seems to be a promising molecular marker related to the EMT in EC [52]. High micro-RNA 21 expression levels in EC seem to play an important role in PTEN’s downregulation, which leads to EC cell proliferation [63]. MicroRNAs (miRNAs) are small non-coding RNA molecules, and they can affect multiple target genes involved in modulating the EMT with an impact on CSC properties through targeting the PTEN-PI3K-AKT-mTOR axis. Targeting the key signaling components of the PI3K/AKT pathway by restoring miRNA could be a promising therapeutic approach to suppressing the EMT, thus targeting the PI3K/AKT pathway in endometrial cancer [57]. MicroRNAs (miRNAs) play a particularly important role in gene expression. Exosomes containing miRNAs mediate communication between EC cells, fibroblasts, and tumor-associated macrophages (TAMs), thus leading to tumor microenvironment (TME) formation. Oncogenes carried by exosomes can induce the malignant transformation of target cells [64].

Immune cells also regulate CSCs, and there is cross-talk between them, thus resulting in immune evasion in the TME, which substantially contributes to tumor progression [54]. Tumor-associated macrophages (TAMs) from the cancer microenvironment play a key role in EC progression, with clinical relevance in targeted therapy. The CHD4 R975H mutation revealed in endometrial CSCs profoundly impacts several stemness signaling pathways, including mTOR, TNF-α signaling via NF-κB, and KRAS, and growth factor signaling induces the M2-like polarization of tumor-associated macrophages (TAMs) and, subsequently, stemness in EC cells [65]. Increased levels of pro-tumorigenic macrophage factors in a supernatant collected from a CSC sphere culture have been found, such as those of IL-13 and TGF-β [66]. The incubation of TAMs with such a sphere culture showed that macrophage polarization tended toward an immunosuppressive phenotype [54,67,68].

Additionally, TAMs induce the secretion of soluble mediators, such as IL-6, TGF-β, and WNT ligands, which leads to them influencing CSC phenotypes [67]. TAMs have a direct influence on CSCs, thus activating NF-κB, and sustain the stem cell state of CSCs [54]. This has a clinical implication as, for example, the inhibition of IL-6 produced by TAMs with tocilizumab prevents the generation of CD44^+^ cells [54,69]. 

A protein involved is maternal embryonic leucine zipper kinase (MELK), which mediates various cascades of signal transduction, thus regulating the tumor microenvironment (TME). This process affects the response of immune cells to regulate tumor progression, and it plays a key role in tumor cell apoptosis, proliferation, invasion, and metastasis. MELK is a member of the AMPK (AMP-activated protein kinase) protein family, which is expressed in many malignancies. Excellent results have been obtained in clinical trials with inhibitors developed to target MELK. The molecular mechanism of MELK in the process of cancer evolution is not completely known [70].

Diverse proteins have implications in the CSC stemness process. Recently, PD-L1 has also been shown to be involved in the self-renewal of cancer stem cells. PD-L1 has an influence on oncogenes related to stemness expression, such as aldehyde dehydrogenase 1 (ALDH1), OCT4, NANOG, and SOX2, as well as on surface stemness markers, such as CD133. PD-L1 knockdown inhibited endometrial CSC tumorigenicity dependent on hypoxia HIF-1αα and HIF-2αα activation, which came with clinical implications in targeted therapy using PD1/PD-L1 immunotherapy [71].

Different stemness markers observed in EC in three-dimensional cell cultivation specimens, such as high levels of aldehyde dehydrogenase (ALDH), are associated with stemness pathways, such as the glycolytic pathway, angiogenesis in tumors, the activation of hypoxia, and poor survival outcomes [72]. GLUT1 inhibition synergized with ALDH inhibition can block endometrial cancer proliferation, which has clinical importance for therapeutic decision-making and prognostic evaluation [72].

Metabolic syndromes, such as Diabetes Mellitus and dyslipidemia, increase the risk of drug resistance in EC [73]. CSCs undergo metabolic adaptations to facilitate their proliferation, invasion, and metastasis. Aerobic glycolysis (Warburg effect) is the main energy provider in EC cells, especially in CSCs, while oxidative phosphorylation (OXPHOS), another metabolic form of energy, is reduced or impaired. In this regard, agents targeting the glycolysis can inhibit cancer cell proliferation. For example, metformin, which is a specific medication used in hyperglycemia and in weight control through diet, reduces the incidence of EC and improves the prognosis of EC patients [73].

**Figure 2:**
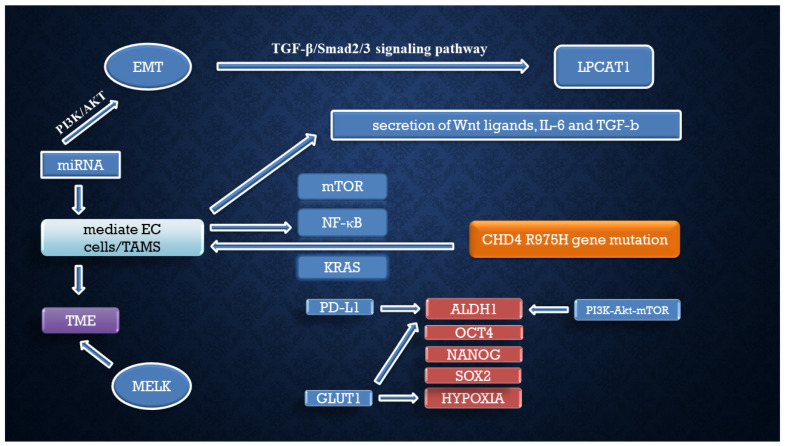
Secondary stemness pathways in endometrial cancer.

The activation of the PI3K-Akt-mTOR pathway has an important role in aggressive phenotypes of EC. Aldehyde dehydrogenase (ALDH) has an influence on mTORC1 through retinoic acid-induced lactate dehydrogenase A (LDHA) activation. The combination of aldehyde dehydrogenase (ALDH) and PI3K-Akt inhibitors has a higher impact on the proliferation of endometrial cancer as it was found to reduce endometrial cancer cell growth. Targeting the PI3K-Akt-mTOR pathway along with ALDH-influenced glycolysis could play a pivotal role in identifying novel strategies for the management of this aggressive cancer [74] (Table 1). Secondary stemness pathways are highlighted in Figure 2.

## 5. Treatments Based on Targeting Stemness Pathways and Future Directions

Targeting CSCs is of great interest for improving the prognosis of patients with EC. Potential therapeutic targets are currently being sought, including signaling pathway inhibitors, antiangiogenic agents, selective estrogen receptor downregulators, poly (ADP-ribose) polymerase (PARP) inhibitors, and immune checkpoint inhibitors [7]. Immunotherapy targeting programmed death cell protein 1 PD-1 and its ligand PD-L1 in EC is a promising therapeutic approach to preventing recurrence since PD-L1 expression is involved in maintaining the stemness of CSCs [61]. Since high ALDH1A1 expression levels were associated with poor survival, ALDH inhibition may serve as a new clinical treatment option for endometrial cancer [75]. Metformin inhibited ALDH, but future studies should investigate its role as an adjuvant therapy in EC [76]. Along with metformin, dietary compounds and microRNAs may be promising in targeted therapies via autophagy modulation in cancers [77].

Selective inhibitors of PI3K and mTOR, such as Bimiralisib, have been studied in preclinical models and clinical trials. This is the first study to reveal the positive effects of a PI3K/mTOR dual inhibitor on endometrial cancer cell lines [78]. Another mechanism is the use of long non-coding RNAs (lncRNAs), which modulate CSC characteristics via epigenetic, transcriptional, and post-transcriptional regulation [79,80,81,82]. The suppression of lncRNAs was found to inhibit the self-renewal, proliferation, migration, and invasion of CSCs [79]. The combination of gedatolisib (a pan-class I PI3K/mTOR isoform inhibitor) and PTK7-ADC (an antibody drug conjugate used against the cell-surface tyrosine-protein kinase 7) has a double synergistic effect on advanced solid tumors [79].

Researchers have conducted clinical trials targeting mTOR signals in EC [83]. Rapamycin, a representative mTORC1 inhibitor, is considered a drug that acts against CSCs [83,84]. Metformin is another drug that inhibits the mTOR pathway through the activation of adenosine monophosphate-activated protein kinase (AMPK) [85,86,87], a reduction in the levels of the CD44 stemness marker [88], and VEGF and TGFβ1 downregulation [89]. Another mTOR inhibitor, gedatolisib (RAD001) [83,87], induces apoptosis in cancers by upregulating the expression levels of genes responsible for apoptosis [83,90]. Other studies showed that ropivacaine inactivated the PI3K/AKT signaling pathway in cancers and accelerated cell ferroptosis [91]. A novel LIFR inhibitor, EC359, was developed to induce apoptosis in EC cells, with a significant impact on the AKT/mTOR pathway. It also reduced the levels of cancer stem cell markers OCT4, SOX2, and NANOG [92], polarizing tumor-associated macrophages (TAMs) toward the M1 phenotype [93]. Bimiralisib (PQR309), an orally selective inhibitor of PI3K and mTOR, has been studied in clinical trials, exhibiting a positive feedback loop in endometrial cancer cell lines [94].

Novartis developed LGK974, which acts against the Wnt signaling pathway. The inhibition of porcupine, required for Wnt activation and secretion, was induced by LGK974 (Novartis, Basel, Switzerland; https://www.novartis.com), which reduced the expression levels of WNT target genes in preclinical models [20]. Medroxiprogesterone acetate has a tumorigenic effect on early endometrial carcinogenesis by inhibiting Wnt/β-catenin signaling gene expression [7,95]. An intrauterine device with levonorgestrel showed similar efficacy [28]. DKN-01 is a humanized monoclonal antibody (Mab) that was developed as an anti-neoplastic agent inducing Wnt signaling genetic alterations in recurrent epithelial endometrial cancer [28]

Wnt-driven cancers can be targeted with porcupine inhibitors, which overcome the limitations of β-catenin inhibitors [28]. Niclosamide, which is used in the treatment of tapeworm infections, targets the Wnt/β-catenin pathway [96,97,98]. Niclosamide in combination with Sorafenib was shown to modulate cancer stemness, and IGF-1R/p-IGF1R/OCT4 had strong properties in terms of stemness and the epithelial–mesenchymal transition with synergic effects [99]. The FDA approved the antiparasitic drug Ivermectin in anticancer therapy through Wnt inhibition [100], along with Niclosamide [96]. Salinomycin, an antibiotic that interferes with Wnt/β-catenin signaling, induces apoptosis in cancers [26,28,96] and causes the apoptosis of cells [19]. Salinomycin inhibits several stemness pathways, such as PI3K, AKT, Wnt/β-catenin, and mTOR, with relevance in cancer therapy [101]. Darifenacin, a novel muscarinic receptor 3, has been shown to be a potential WNT inhibitor in anticancer studies [96,102]. Tolfenamic acid, an anti-inflammatory drug, induced the degradation of β-catenin [103]. Natural polyphenols such as quercetin and resveratrol could inhibit WNT/β-catenin, thus being potential candidates for the discovery of anticancer drugs [104]. Sesamolin could be a valuable natural drug for EC therapy, targeting the Wnt pathway synergistically with chemotherapy and endocrine [105].

NF-κB inhibitors were found to have low efficacy in lymphoma and leukemia [106,107] treatment, but in combination with other chemotherapies, they can be as useful as most anticancer agents [108]. Thalidomide has an NF-kB inhibitor effect [109,110], but its use increases the risk of thrombotic events [111]. Bortezomib is a protease inhibitor, and it has an anticancer effect mediated by NF-kB [20]. Estradiol and estrone also have a significant impact on the NF-kB pathway, the EMT, and, subsequently, on stemness [106]. Another targeting mechanism of CSCs is the inhibition of the EMT. Sigmasetrol, a natural phytosterol showing such an effect, reduces the expression of cancer stemness genes in EC cells and suppresses β-catenin, OCT 4, and the mTOR pathway in EC [112].

The Notch signaling pathway can be targeted by γ-secretase inhibitors (GSIs) and monoclonal antibodies against Notch ligand receptor interactions (mAbs). In combination with conventional therapies, these inhibitors can be effective [20] in terms of stem cell marker expression, tumor growth, angiogenesis, and metastasis [33,35]. Several monoclonal antibodies have been used as inhibitors of Notch ligands (DLL-4) and receptors (Notch1–3) [33,35]. Enoticumab is an antibody against DLL-4 that seems to be safe and efficient in advanced solid cancers. Demcizumab, which is a DLL-4 antibody, did not improve efficacy, which is why it is not in clinical development [20,33,35].

In targeting Hedgehog, Hh, pathway inhibitors, efficacy is dependent on the level of alterations in this signaling cascade [20]. SMO and Gli transcription factors, which are proteins that participate in the signaling cascade, are the main targets currently being studied [113]. Cyclopamine is an SMO inhibitor with significant side effects, which prevent its use in humans. Sonidegib is another SMO antagonist, which was approved in 2015 by the FDA. Arsenic trioxide is a Gli inhibitor approved by the FDA for promyelocytic leukemia treatments [114]. Genistein (phases I and II), which is an isoflavone that inhibits Gli, can be used against CSCs [20,115]. We present these treatment options in a table (Table 2).

Another treatment option is the use of ROS scavengers. Cancer stem cells possess a lower ROS level compared with non-tumoral stem cells through different mechanisms, such as the Warburg effect, that allow them to maintain stem cell properties. The Warburg effect is characterized by the switch from aerobic to anaerobic glycolysis regardless of the oxygen level in the microenvironment [20,116,117,118]. For this reason, the possibility of ROS scavengers being used as an anticancer strategy depends on the tumor capacity to modulate its metabolism [20,118,119,120].

Regarding future directions, researchers are concerned with the clinical application of knowledge of the various stemness pathways involved in endometrial cancer. New treatment options have been sought out in the effort to eradicate EC, such as ALDH inhibitors [75], and the use of microRNAs may serve in autophagy modulation in cancers [77]. Also, the described stemness pathways should be targeted as new cancer treatment options, and new efforts are being made to reveal the positive effects of a PI3K/mTOR dual inhibitor on endometrial cancer cell lines [78,79,91]. Also, mTOR inhibitors were shown to have promising results, regulating genes involved in apoptosis [82,83,87,90]. Compounds used in the treatment of tapeworm infections showed efficacy in targeting the Wnt/β-catenin pathway [96,97,98,99,100]. Natural drugs could inhibit WNT/β-catenin, thus being potential candidates for the discovery of anticancer drugs [104]. NF-kB inhibitors [109,110], Hedgehog (Hh), pathway inhibitors, and Notch ligand receptor inhibitors are also under investigation [20]. As a new direction in clinical trials, starting with the importance of hypoxia in the stemness properties of EC, ROS scavengers are investigated, but their use in anticancer management depends on the tumor metabolism [20,118,119,120]. A novel therapeutic avenue is based on Wilms tumor 1-associated protein/early growth response factor 1/phosphatase and tensin homolog (WTAP/EGR1/PTEN) pathway targeting, which is involved in the chemotherapeutic resistance of EC [121].

In our review, we focused on stemness pathways, but stemness-related genes are also involve, such as NANOG (Nanog homeobox), OCT4 (octamer-binding protein 4), and SOX2 (sex-determining region Y-box 2) [122]. Machine learning techniques can identify prognosis-associated genes in patients with EC [123]. A robust prognostic model can be developed based on RNA binding proteins that allow us to identify individualized diagnosis and treatment [124].

It was found that the CHD4 R975H mutation is promoting a cancer stem cell (CSC)-like phenotype by the activation of signaling pathways, such as NF-κB, mTOR, KRAS, p53, and TGF-β. It also induces M2 polarization of TAMs. Valuable insights into the molecular mechanism of EC have been obtained [125].

Also, the surface markers CD133^+^ and CD44^+^ can be useful as predictive biomarkers for prognosis in endometrial cancer. Targeted treatments can be developed against these surface markers [122,126]. There are studies emphasizing the crucial roles of CD44, FGF2, and orMMP2 in EC stemness and their potential as biomarkers, especially therapeutic targets in this pathogenesis [127], while other researchers have highlighted the value of Sonic Hedgehog (SHH) pathway expression as a robust prognostic biomarker, also used for tailored therapeutic management in EC [128].

## 6. Conclusions

Targeting stemness pathways is a promising strategy to eradicate tumors and metastasis. CSC phenotypes are regulated through many pathways, which provide mechanistic support for drug resistance. Some of these stemness pathways and their targeting approaches have been summarized in our review. CSCs’ inhibitory activity in specific targeted pathways depends on the extent of their molecular effects. The aim of a targeted treatment is to reduce CSCs’ undesirable toxicity by preventing them from targeting normal cells.

In our review, we observed a strong interconnection between stemness pathways that regulate CSC phenotypes and may lead to resistance in single pathway inhibitors. Advanced tumors also contain mostly polyclonal cancer stem cells, which can enable them to have therapy resistance against single pathway inhibitors.

Despite these challenges, many inhibitors are being tested in clinical trials. Researchers are aiming to develop less toxic compounds targeting stemness pathways and combine compounds to overcome the above-mentioned challenges.

## Figures and Tables

**Table 1:** Stemness pathways and their activities and interconnections.

References	Pathway	Activity	Connections
[20,21,22]	Hippo	Influence apoptosis Regulate autophagy Regulate detoxification	Wnt
[1,23,71,72]	Nanog	Stem cell dormant state preservation	OCT 4, SOX2, PD1-PD-L1, ALDH
[1,8,20,24,25,26,28]	Wnt signaling pathway	Proliferation, invasion, metastasis	Hedgehog Notch
[30,31,32]	NF-kB	Proliferation, differentiation, inflammation, immune system response	
[1,20,36]	Notch	Stem cell dormant state preservation Function as oncogene	Wnt
[20,40,41]	Hedgehog	Cell proliferation and adhesion	Wnt EMT
[20,42,43,48,73]	TME, hypoxia	Cancer initiation, abnormal CSC differentiation, DNA and RNA modification, protein and lipid alteration, cancer cell resistance to ROS, proliferation, invasion, apoptosis evasion, angiogenesis	Nanog, OCT4, SOX2, Notch Wnt, NF-kB
[1,52,53,62]	EMT	Proliferation, invasion, decrease adhesion	TME, hypoxia Notch Metabolic disorder
[1,58,74]	PI3K-mTOR	Upregulation of EMT inducer, histone regulation, adhesion downregulation, invasion, metastasis, cellular plasticity	EMT, epigenetic regulation, ALDH
[1,52,64]	Epigenetic modulator, miRNA	PTEN downregulation, cell proliferation	EMT PI3K-mTOR TAM TME
[54,70]	TAM	Pro-tumorigenic macrophages induce immune evasion	TME, mTOR, NF-kB, Wnt

Legend: octamer-binding transcription factor 4—Oct4; SRY-Box 2—SOX2; aldehyde dehydrogenase—ALDH; NF-kB—nuclear factor kB; PTEN/Phosphoinositide 3 kinase (PI3K)/protein kinase B (AKT)/mammalian target of rapamycin (mTOR)—PI3K-mTOR; tumor microenvironment—TME; Wingless Int-1—Wnt; EMT—epithelial–mesenchymal transition; tumor-associated macrophage—TAM.

**Table 2:** Treatment options targeting stemness pathways.

Manuscript	Treatment	Target
[61]	Immunotherapy	PD-1, PD-L1
[76]	Metformin	ALDH
[78,94]	Bimiralisib	PI3K, mTOR
[79]	Gedatolisib	PI3K, mTOR
[83,84]	Rapamicyn	mTOR
[85,86,87]	Metformin	mTOR
[83,87]	Everolimus	mTOR, PI3K
[92]	LIFR inhibitor, EC359	mTOR
[20]	LGK974	Wnt
[7,95]	Medroxiprogesterone acetate	Wnt
[28]	Levonorgestre	Wnt
[96,97,98]	Niclosamide	Wnt, EMT
[96]	Mebendazole, Albendazole	Wnt
[26,28,96]	Salinomycin,	Wnt/beta-catenin
[101]	Salinomycin,	mTOR, PI3K, Wnt
[96,102]	Darifenacin	Wnt
[103]	Tolfenamic acid	β-catenin
[104,105]	Quercetin, Resveratrol, Sesamolin	Wnt
[109,110]	Thalidomide	NF-kB inhibitor
[20,33,35]	Enoticumab, Demcizumab	Notch
[20]	Bortezomib	NF-kB inhibitor
[112]	Sigmasetrol	mTOR, β-catenin
[20,113]	Cyclopamine	Hedgehog
[20,115]	Genistein	Hedgehog

Legend: programmed death cell protein 1 and its ligand PD-1—PD-L1; aldehyde dehydrogenase—ALDH; PTEN/Phosphoinositide 3 kinase (PI3K)/protein kinase B (AKT)/mammalian target of rapamycin (mTOR)—PI3K-mTOR; Wingless Int-1—Wnt; EMT—epithelial–mesenchymal transition.

## Data Availability

No new data were created.
